# The Hygiene Hypothesis and Its Inconvenient Truths about Helminth Infections

**DOI:** 10.1371/journal.pntd.0004944

**Published:** 2016-09-15

**Authors:** Neima Briggs, Jill Weatherhead, K. Jagannadha Sastry, Peter J. Hotez

**Affiliations:** 1 Department of Immunology, University of Texas M.D. Anderson Cancer Center, Houston, Texas, United States of America; 2 The University of Texas Graduate School of Biomedical Sciences at Houston, Houston, Texas, United States of America; 3 Sabin Vaccine Institute and Texas Children’s Hospital Center for Vaccine Development, Departments of Pediatrics and Molecular Virology and Microbiology, National School of Tropical Medicine, Baylor College of Medicine, Houston, Texas, United States of America; 4 James A Baker III Institute for Public Policy, Rice University, Houston, Texas, United States of America; 5 Department of Biology, Baylor University, Waco, Texas, United States of America; 6 Scowcroft Institute of International Affairs, Bush School of Government and Public Service, Texas A&M University, College Station, Texas, United States of America; Universidad San Francisco de Quito, ECUADOR

## Abstract

Current iterations of the hygiene hypothesis suggest an adaptive role for helminth parasites in shaping the proper maturation of the immune system. However, aspects of this hypothesis are based on assumptions that may not fully account for realities about human helminth infections. Such realities include evidence of causal associations between helminth infections and asthma or inflammatory bowel disease as well as the fact that helminth infections remain widespread in the United States, especially among populations at greatest risk for inflammatory and autoimmune diseases.

## Introduction

Beginning in the late 20th century, there was an increase in the reported cases of inflammatory and autoimmune diseases worldwide, such as asthma, inflammatory bowel disease (IBD), food allergies, and multiple sclerosis (MS) [1]. This phenomenon has been linked to the acquisition of a “westernized” lifestyle, declining family size, improved household amenities, higher personal cleanliness, and reduced cross infection within affluent communities [2]. From this notion the “hygiene hypothesis” evolved, suggesting a reduction in microbial exposures, secondary to hygienic conditions, which impede the proper maturation of the immune system. The hygiene hypothesis is accepted by many in the global scientific community and has evolved to associate multiple variables as either protective or contributory in the development of inflammatory diseases. Identified environmental factors that are incorporated into the hygiene hypothesis include variations in microbial exposure, parasites, diet, medications, lifestyle behaviors, sanitation, occupations, and pollutant exposures [1,3]. A branch of this hypothesis points at parasitic helminths—which, according to some, are nearly eradicated from high-income countries—as a key immune modulator necessary for proper immune development. This movement goes so far as to propose redefining the role of certain helminths within the human host from parasites to mutualists and to test their therapeutic potential in humans and animal models [4].

We argue here that parasitic helminth infections have not been demonstrated as necessary for proper host immune maturation in either humans or in animal models. Several animal studies and select human studies have demonstrated beneficial anti-inflammatory responses to autoimmune and inflammatory diseases when treated with helminths. However, a number of other studies have noted helminths can exacerbate and even induce inflammatory conditions. Without significant evidence to support their therapeutic potential, we express concern over the safety of administrating living parasites with known human morbidity. We instead suggest directing future studies towards the identification and trial of key helminth-derived immunomodulatory molecules that might provide a safer and better mechanistically understood therapy [5].

## Global Impact

Over one billion people are infected with parasites globally, with an overrepresentation of infections in people who live in extreme poverty [6]. Helminths, including hookworm (mostly *Necator americanus*), whipworms (*Trichuris trichiura*), roundworms (*Ascaris lumbricoides*), and schistosomes (mostly *Schistosoma haematobium* and *Schistosoma mansoni*) cause chronic, debilitating diseases—which include iron deficiency anemia, asthma, vitamin deficiency, abdominal pain, colitis, and dysentery—leading to growth failure and impaired cognitive development [7]. Schistosomiasis is associated with ulcerative genital disease, infertility, and increased transmission of human immunodeficiency virus [8]. According to the Global Burden of Disease Study 2010, overall helminth infections account for more than 14 million disability adjusted life years (DALYs), ranking these diseases among the greatest global health threats [6]. Helminth infections simultaneously promote poverty within endemic communities because of economic suppression from decreased education accruement, loss of workforce, and cost of treatment [6,9,10].

## Impact of Helminths on Host Inflammation and Atopy

Beyond the public health and economic impact of human helminth infections, there is a profound level of global disability that arises from the pathologic sequelae of helminth infections translating into widespread mucosal dysregulation and contributing to inflammatory diseases, such as asthma and inflammatory bowel disease (Box 1) [11]. While there are limited clinical studies reporting asthma and inflammatory bowel disease from rural, helminth-endemic areas, we believe this is secondary to underreporting of disease because of limited diagnostic resources. An Ecuadorian cohort of over 2,400 children in Esmeraldas province, where 28.6% of children had at least one documented soil transmitted helminth, documented that 25.9% of children had wheeze, 15.2% had skin test reactivity to an aeroallergen, and 17.7% had an episode of eczema, suggesting a significant presence of allergic disease within the community [12].

**Box 1. Proinflammatory Disease Effects of Human Helminths**

Asthma and Loeffler’s pneumonitis

- Ascaris lumbricoides
- Toxocara canis
- Toxocara cati

Inflammatory bowel disease: enteritis and colitis

- Schistosoma mansoni
- Trichuris trichiura
- Strongyloides stercoralis

### Asthma

Ascariasis (*A*. *lumbricoides* infection) is a risk factor for the development of asthma phenotypes in endemic regions [13]. It has been well documented since the early 20th century that *Ascaris* larvae migrate through the lungs during the parasite life cycle, resulting in Loeffler’s pneumonitis, a clinical disease that resembles asthma in humans [11,14]. Pneumonitis secondary to acute *Ascaris* larvae migration is particularly evident in areas where prevalence is low or periodic. For instance, in Saudi Arabia, ascariasis was previously an important cause of seasonal pneumonitis with eosinophilia, in which patients present with cough, dyspnea, and substernal chest pain [15].

In humans, elevation of *Ascaris*-specific immunoglobulin E (IgE), which could indicate either recent or remote *Ascaris* infection, has been shown to be an independent risk factor for asthma phenotypes and is associated with increased asthma disease severity, emergency center evaluation, and crossreactivity to bystander antigens, such as house dust mites [16–18]. Specifically, bronchial hyperreactivity—including increased airway resistance and decreased forced expiratory volume in one second (FEV1) and FEV1/forced vital capacity (FVC) ratio—has been demonstrated in children with elevated *Ascaris*-specific IgE [19,20]. While elevation of *Ascaris*-specific IgE provides a consistent link between ascariasis and asthma phenotypes, conflicting evidence exists for patients with detectable *Ascaris* eggs in the stool. In a study by Palmer et al. conducted in eight rural counties in China, a positive stool exam for *Ascaris* was associated with an increased risk of asthma, indicating that current intestinal ascariasis may also predispose patients to develop an asthma phenotype. Furthermore, patients who had a positive stool exam for *Ascaris* eggs also had increased sensitization to bystander aeroallergens [21]. However, other studies have demonstrated no enhanced risk of asthma in patients with detectable *Ascaris* eggs within the stool at the time of evaluation [18]. Furthermore, several studies have concluded that *A*. *lumbricoides* is not associated with heightened atopic signs and symptoms, such as allergen skin test reactivity and allergic dermatitis, as previously described [22,23]. Studies using anthelminthic treatment in patients with asthma have been met with conflicting results as well. A prospective study of albendazole or praziquantel in a schistosomiasis-endemic area of Brazil did not show any respiratory benefit in children with asthma but led to overall worsening of asthma severity after repeated anthelminthic administration [24]. Conversely, the use of anthelminthic therapy, albendazole, in a cohort of asthmatic patients in an *Ascaris*-endemic region of Venezuela resulted in reductions in inhaled maintenance and rescue therapy as well as a reduction in asthma exacerbations [25]. In the setting of conflicting human studies, mouse models of ascariasis have been developed to evaluate the association between ascariasis and asthma.

*A*. *suum*-infected mouse models—a similar species to *A*. *lumbricoides* and a human pathogen—are currently being used to uncover the mechanism by which *Ascaris* is linked to pneumonitis and asthma phenotypes [26]. In a recent study by Nogueira et al., repetitive infection with *A*. *suum* was found to cause chronic lung parenchyma damage, including inflammatory infiltration consisting of neutrophils and eosinophils, thickened intralveolar septa, and evidence of tissue remodeling mimicking the pathologic changes noted in asthma. Functionally, the *A*. *suum*-infected mice have reduced dynamic airway compliance as well as FEV1 and FVC consistent with an asthma phenotype [27]. Additionally, *A*. *suum*-infected mice have expansion of type 2 innate lymphoid cells (ILC2) and tissue-resident T_h_2 cells in the lungs. Proliferation of these cell lines are thought to reduce helminth worm burden upon reinfection. However, after helminth infection, the presence of ILC2 and T_h_2 cells in the lungs promote massive airway type II inflammation when challenged with intratracheal house dust mite, a common allergen. This suggests that helminth infection may sensitize the mice to other environmental allergens [28]. Furthermore, after antigen challenge, recruitment of antigen-activated CD4^+^ T cells and eosinophils expressing high levels of cell surface marker CD44 to the lungs has been shown to play a critical role in the regulation of pulmonary inflammation and asthma. Similarly, administration of *A*. *suum* extract intranasally promotes eosinophil and CD4^+^ T cell accumulation in the lungs as well as an increase in airway resistance after methacholine challenge in a CD44-, hyaluronic acid–dependent mechanism in a mouse model [29].

While conflicting data is present within the literature, it is evident that ascariasis adversely affects the integrity of the lungs and host inflammatory and immune responses, causing increased airway resistance similar to asthma. Given that ascariasis is one of the most common affliction of children and adults living in poverty, affecting more than 800 million people, presumably millions of these individuals are potentially at risk of developing significant pulmonary morbidity secondary to ascariasis [6]. Thus, further studies to elucidate the mechanism involved in ascariasis and asthma are warranted.

### Inflammatory Bowel Disease

Trichuriasis (a disease infecting over 400 million people that results from infection by the human whipworm [*T*. *trichiura*]) and intestinal schistosomiasis (a disease infecting over 250 million people that results from infection by *S*. *mansoni*) are major causes of pathology of the small and large intestine [6,30]. *T*. *trichiura* attaches to the colon, where it can induce acute and chronic colitis, *Trichuris* dysentery syndrome (TDS), and rectal prolapse, especially in heavy infections [31,32]. Long standing trichuriasis, like with IBD, leads to growth suppression and cognitive impairments [33,34]. These sequelae can resolve after treatment, sometimes resulting in catch-up growth [35].

As opposed to the T_h_2-mediated expulsion of *T*. *muris* seen in resistant mouse strains, mouse models of chronic trichuriasis result in massive crypt hyperplasia because of intra-epithelial lymphocytosis in the large intestines, hypothesized to be driven by a parasite-derived interferon gamma (IFN-γ) homologue [36,37]. MUC5AC mucin, which is normally expressed in mucosa of the intestines and airway, was shown to be up-regulated in both *T*. *muris* infections and human ulcerative colitis, and blocking of ectopic MUC5AC expression was identified as a likely contributor in the effectiveness of oral tacrolimus therapy in patients with refractory ulcerative colitis [38,39]. Furthermore, recent studies have shown that *T*. *muris*-infected colonic tissue histologically resembles established mouse models of IBD, with a defective epithelial barrier and a dominant T_h_1 immune infiltrate, leading to impaired intestinal mucosa homeostasis [40]. Resistin-like molecule (RELMβ)—a protein that is secreted from goblet cells after local tissue damage triggers a proinflammatory cytokine milieu, including IFN-γ and tumor necrosis factor-alpha (TNF-α)—is thought to be driving the T_h_1 immune infiltration, leading to the chronic colonic pathology in both trichuriasis and IBD [41,42]. Expanding on their comparative analysis of *Trichuris*-induced colitis and IBD, in 2013, Levision and colleagues identified through quantitative trait loci mapping several key overlapping genes expressed during human Crohn’s disease and *Trichuris*-induced colitis [43]. Interleukin 18 (IL-18), a key immune regulator of intestinal homeostasis, was shown to be overexpressed in the human large intestine during both Crohn’s disease and in *T*. *muris*-infected mice [44–46]. Importantly, IL-18 production in *T*. *muris*-infected mice showed direct suppression of critical T_h_2 cytokines, IL-4 and IL-13, necessary for worm expulsion [44]. More recently, Nowarski and colleagues showed that overexpression of IL-18 led to significant mucosal barrier dysfunction, including epithelial goblet cell hyposecretion of protective mucins and other essential proteins for barrier integrity [47]. The clinical, immunological, and histological homology, as well as genetic susceptibility, between *Trichuris*-induced colitis and both Crohn’s disease and ulcerative colitis warrants further investigation of *Trichuris*-induced colitis as an environmental driver of IBD worldwide. There is a need to conduct further epidemiological studies in order to determine the attributable risk of colitis in low- and middle-income countries to trichuriasis.

## Counterpoint: Immunomodulation

Despite the severe negative consequences of parasitic infections that are highlighted above, some studies have presented a counterview of generalized human helminth infections as potentially protective against the development of autoimmune and inflammatory diseases. This protection is hypothesized to be due to their known immunomodulatory properties within a host. Helminths can maintain host evasion for years through an array of mechanisms that down-regulate host innate and adaptive responses. These include immunomodulatory proteins that contain host-related glycans and lipids that direct cytokine mimicry and interference, nonprotein signature molecules that result in immunosuppressive host cytokine release, and direct interference of antigen presentation. The presence of T regulatory cells (Treg) along with immune modulatory cytokines IL-10, TGF-β, and, at basal levels, IL-18, promote helminth survival while simultaneously quelling the host inflammatory response [44,48]. One example of helminth-induced immunosuppression is the rodent parasite *Heligmosomoides polygyrus bakeri*, which is hypothesized to recruit Tregs to control colitis, in part through secretion of IL-10. Additional studies have shown other, phylogenetically distinct helminthic species—including *Hymenolepis diminuta* (cestode), *S*. *mansoni* (trematode), and *Litomosoides sigmodontis* (nematode)—are capable of reducing inflammation in the gut, predominately through IL-10 secretion[49].

## Helminths as Causes of Atopy and Asthma in High Income Countries

We are left with two seemingly contradictory sets of findings regarding the role of helminth parasites in human inflammatory disease and atopy. A recent *Lancet Infectious Diseases* review questioning helminth therapy versus elimination by Wammes and colleagues highlights this question by identifying the discrepancy between findings in murine models and human studies [5]. Even several large-scale randomized human trials on the effects of helminths on allergic diseases show contradictory results [5].

Adding to the confusion of these findings is an erroneous assumption on which a component of the hygiene hypothesis is sometimes based: namely, that helminth infections have largely disappeared in high-income countries. A 2011 systematic review by Starr and Montgomery identified a now 30-year gap since the last high-quality epidemiological study on soil-transmitted helminths (hookworm, *A*. *lumbricoides*, *T*. *trichiura*, and *Strongyloides stercoralis*) in the United States [50]. In combination with 13 other high-quality studies, the review notes that helminths afflicted more than 50% of people in the southern US and Appalachia as recently as 1982 [50]. They conclude that the current prevalence of disease in the US remains unclear and that there is a need for new studies [50]. Without more recent studies on soil-transmitted helminths, human toxocariasis—a zoonotic helminth infection from dogs and cats caused by *Toxocara canis* and *Toxocara cati* respectively—is considered to be the most common helminth infection in the US [51,52]. According to the Centers for Disease Control and Prevention (CDC), more than 20% of African Americans living in poverty—a group that is considered at highest risk for asthma and other atopic diseases—are seropositive for *Toxocara* infection [53]. Indeed, there are several important studies that show *Toxocara spp*. elicit Loeffler’s type pathology during their larval migrations through the human lungs, causing an asthma phenotype [11,54,55]. A large population-based study conducted in the US demonstrated that patients with seropositivity to *Toxocara* had decreased FEV1 when additional population-based factors such as age, sex, and body mass index were controlled [56]. Thus, rather than protect against asthma, helminths today may represent an environmental cause of inflammatory disease in the US [51,57].

## Iatrogenic Helminth Infections: A Good or a Bad Idea?

Given the contradictory findings highlighted above, it is not surprising that the iatrogenic administration of helminths—labeled as a safe, efficacious treatment strategy for inflammatory and autoimmune diseases—has met with mostly mixed or negative results.

For asthma or allergic rhinitis, there appears to be no benefit from administration of parasitic helminths. Specifically for asthma, in a randomized control trial, no benefit of hookworm larval administration was observed [58]. Additionally, three randomized trials, one using *T*. *suis* eggs [59] and the other two using hookworm [58,60], failed to show any therapeutic benefit against grass pollen–induced allergic rhinitis, allergic rhinoconjunctivitis, or asthma. In fact, in two of these three trials, patients suffered from gastrointestinal symptoms as a result of the helminth infection. An additional Cochrane review evaluating the efficacy of helminth therapy for allergic rhinitis management found that participants receiving helminth therapy showed no improvement in rhinitis symptoms or number of well days but did have increased adverse events, including local pruritus and gastrointestinal symptoms such as abdominal pain, flatulence, and diarrhea [61].

For IBD and celiac disease, therapy has focused on using the pig whipworm *T*. *suis* or the human hookworm *Necator americanus*. Regarding the former, two open-label studies by Summers et al. showed that *T*. *suis* therapy for the treatment of IBD correlated to a remission rate of around 70%, helping ignite the excitement of helminth therapy in human beings [62,63]. In the follow-up placebo-controlled, double-blind, randomized trial, there was an improvement in ulcerative colitis disease severity but no significant improvement in the rates of remissions [64]. Two subsequent Phase 2 clinical trials in the US, with 250 patients (TRUST-1, trial identifier NCT01576471), and Europe, with 240 patients (FALK, trial identifier NCT01279577), using *T*. *suis* eggs for moderate-to-severe Crohn’s disease were terminated in 2013 because of a lack of efficacy, which was determined by both a measure of disease activity index and remission rates [65,66]. A 2014 Cochrane review that evaluated the safety and efficacy of *T*. *suis* therapy to induce inflammatory bowel disease remission found insufficient evidence, secondary to small studies and low-quality evidence, to support the use of helminth therapy [67]. Although these trials do not show significant short or long-term adverse events to *T*. *suis* therapy, when given up to 7,500 ova, there are continued concerns of safety. Of particular concern is aberrant migration, which is well known to occur with many helminth species when in unnatural hosts [68]. Additionally, zoonotic helminths have been noted for their propensity to be particularly inflammatory when in humans [69]. These concerns, along with limited efficacy, have influenced investigators to explore human helminth-derived molecules and *N*. *americanus*, which has a well-defined natural lifecycle within humans [68,70]. For Crohn’s disease, after 45 weeks of *N*. *americanus* infection, one study showed no statistically significant decrease in disease activity index among patients [71]. A second study using *N*. *americanus* larvae for the treatment of 20 patients with celiac disease showed a reduction in IFN-γ and IL-17 inflammatory cytokines in duodenal biopsies but no improvements in clinical symptoms among those with hookworm treatment [72]. More recently, in a preliminary study, combination therapy of *N*. *americanus* and gluten microchallenge showed improved gluten tolerance in eight celiac disease patients in an open-label, nonplacebo controlled study (66). It was further suggested that hookworm challenge infections might exert such effects by reversing microbial dysbiosis [73]. Although the authors of the celiac study acknowledge that conclusions from the study are preliminary because of the sample size and lack of controls, they argue that further investigation into combination therapy should be considered [74].

Helminth therapy has been tried as a treatment for MS after the observation was made that 12 patients with MS and concomitant natural intestinal helminth infection in Argentina had a reduction in the number of relapses and lower MRI activity in comparison to uninfected MS patients [75]. Building on this promising finding, a Phase 1 clinical trial of *T*. *suis* egg was administered to 5 MS patients during a three-month period and showed a mild reduction in the number of new gadolinium-enhancing lesions, but there were no changes in neurological symptoms, nor was there a placebo-controlled comparison [76].

The failed therapeutic potential of helminth treatment in humans has been surprising to some scientists. As highlighted above, there have been several animal studies that demonstrate an immunosuppressive role of helminths. Further, human epidemiological studies have suggested a protective role for helminths against autoimmune and inflammatory conditions. However, many of these studies identified delayed or diminished development of allergic or autoimmune responses when helminth treatment was used prophylactically, with few studies that demonstrate a clear therapeutic advantage in already established conditions [77]. Furthermore, nearly all of the animal studies used helminth doses significantly higher than would be viewed as safe in humans, which perhaps explains why successful trials in humans were limited [78].

## Conclusion

The rationale for iatrogenic administration of helminths to treat inflammatory diseases has occasionally been based on specious assumptions. Significantly, the assumptions that helminths infections are negligible in the US and other Western nations and that helminths have intrinsic anti-inflammatory properties in humans. We do not argue that helminths evolved with an extraordinary ability to manipulate host immune responses, but their role in relation to humans is not one of symbiosis, as is seen with the bacterial microbiome, but is one of clear parasitism. Given the known risks of live helminth inoculations, research highlighting helminth immunomodulatory properties should direct future research towards identifying helminth-derived molecules of therapeutic potential. This approach is likely to result in a more viable therapeutic window. Further, it’s important not to diminish the role of helminths in inciting and exacerbating inflammatory diseases globally, including in wealthy countries such as the US.

A recently emerged attractive alternative hypothesis to explain the rise of inflammatory diseases is a “biome depletion” theory. This suggests inflammatory disease may be due to a loss of species diversity or alteration of composition of the commensal microbiome within the human body [79]. Interestingly, differences in microbiota composition and diversity between adults who live in high-income versus low- and middle-income communities have been described [80].

The immense conflicting data regarding the benefits versus harms of live helminths as a therapeutic modality to date warrants further questioning of the utility of additional human clinical trials. Therefore, directing future research and trials towards helminth-derived immunomodulatory molecules allows for safer and better-described therapies that could alleviate the suffering from autoimmune conditions without the commensurate risk of a parasite infection. Indeed, the aim of experimental animal models should be to develop novel treatments that mimic the effects of helminths without requiring the presence of parasites in the host.

There is overwhelming evidence that demonstrates the clinical and economic ramifications of helminths in endemic settings [81]. Therefore, prioritizing helminth elimination efforts globally through mass drug administration and developing new control tools, such as anthelminthic vaccines, remains paramount [81].
